# Impact of the CFTR-Potentiator Ivacaftor on Airway Microbiota in Cystic Fibrosis Patients Carrying A G551D Mutation

**DOI:** 10.1371/journal.pone.0124124

**Published:** 2015-04-08

**Authors:** Cédric Bernarde, Marlène Keravec, Jérôme Mounier, Stéphanie Gouriou, Gilles Rault, Claude Férec, Georges Barbier, Geneviève Héry-Arnaud

**Affiliations:** 1 EA 3882-Laboratoire Universitaire de Biodiversité et Ecologie Microbienne, Université de Brest, Brest, France; 2 Centre de Perharidy, CRCM mixte, Roscoff, France; 3 UMR1078, Institut National de la Santé et de la Recherche Médicale, Brest, France; 4 Département de Bactériologie-Virologie, Hygiène et Parasitologie-Mycologie, CHRU Brest, Brest, France; 5 Laboratoire de Génétique Moléculaire, CHRU Brest, Brest, France; 6 Faculté de Médecine et des Sciences de la Santé, Université de Brest, Brest, France; 7 Etablissement Français du Sang—Bretagne, Brest, France; Queens University Belfast, IRELAND

## Abstract

**Background:**

Airway microbiota composition has been clearly correlated with many pulmonary diseases, and notably with cystic fibrosis (CF), an autosomal genetic disorder caused by mutation in the CF transmembrane conductance regulator (CFTR). Recently, a new molecule, ivacaftor, has been shown to re-establish the functionality of the G551D-mutated CFTR, allowing significant improvement in lung function.

**Objective and Methods:**

The purpose of this study was to follow the evolution of the airway microbiota in CF patients treated with ivacaftor, using quantitative PCR and pyrosequencing of 16S rRNA amplicons, in order to identify quantitative and qualitative changes in bacterial communities. Three G551D children were followed up longitudinally over a mean period of more than one year covering several months before and after initiation of ivacaftor treatment.

**Results:**

129 operational taxonomy units (OTUs), representing 64 genera, were identified. There was no significant difference in total bacterial load before and after treatment. Comparison of global community composition found no significant changes in microbiota. Two OTUs, however, showed contrasting dynamics: after initiation of ivacaftor, the relative abundance of the anaerobe *Porphyromonas* 1 increased (p<0.01) and that of *Streptococcus* 1 (*S*. *mitis* group) decreased (p<0.05), possibly in relation to the anti-Gram-positive properties of ivacaftor. The anaerobe *Prevotella* 2 correlated positively with the pulmonary function test FEV-1 (r=0.73, p<0.05). The study confirmed the presumed positive role of anaerobes in lung function.

**Conclusion:**

Several airway microbiota components, notably anaerobes (obligate or facultative anaerobes), could be valuable biomarkers of lung function improvement under ivacaftor, and could shed light on the pathophysiology of lung disease in CF patients.

## Background

With the advent of high-throughput DNA sequencing, the human body can be depicted as a niche of complex microbial communities. Even lungs, long considered sterile, exhibit unexpected microbial diversity, including in healthy people [1–4]. The composition of the airway microbiota is generally disrupted in patients suffering from respiratory disease, especially when there is an infectious component, as in cystic fibrosis (CF) [1,5,6]. Recent studies of CF airway microbial communities depicted a very complex and abundant microbiota [7–9]. Moreover, several fractions of these microbial communities, and variations in their relative abundance, correlated with CF patients’ clinical status [10]. These new findings thus demonstrated the importance of studying the microbiota as a whole rather than focusing on a few well-known pathogens [10,11]. Under this new paradigm, it is important that future clinical studies of respiratory status in CF patients should considered the airway microbiota in their clinical outcome datasets.

Over 1,900 mutations have been reported in the cystic fibrosis transmembrane conductance regulator (CFTR), the gene that is defective in CF patients [12]. Some of these mutations lead to a gating defect of the CFTR protein located in the apical membrane of epithelial cells; one such is G551D-CFTR, which, while a less common mutation in CF patients (approx. 5% of cases), has a very severe clinical phenotype. Recently, a first-in-class molecule named ivacaftor (VX-770) has been shown to increase the activity of wild-type and defective cell-surface CFTR *in vitro* [13]. This CFTR potentiator was tested on CF patients bearing at least one G551D-CFTR allele, and gave very promising results on major clinical parameters (notably, percentage of predicted forced expiratory volume in 1 second: FEV-1), with significant improvement in lung function [14–16]. Because the physicochemical microenvironment influences CF airway microbiota composition [17], it may be hypothesized that ivacaftor treatment, by inducing changes in ion flux and improving ventilation capacity, leads to a shift in microbial communities. Furthermore, ivacaftor has been shown to have antibacterial properties [18], which may also contribute to modifications in microbiota structure. To date, clinical studies of ivacaftor’s effects have mainly focused on conventional clinical and paraclinical parameters [14–16].

The aim of the present study was to analyze in depth the evolution of the airway microbiota in CF patients treated with ivacaftor, using 16S rRNA pyrosequencing to provide as extensive a view of microbial community composition as possible. We closely followed up three CF children attending the same CF centre and receiving standardized medical care. For each patient, at least six sputum samples were collected over a mean 13-month period, ensuring close monitoring over a long period of time, beginning before and continuing after initiation of ivacaftor. Total bacterial load was measured on quantitative PCR, and bacterial communities were analyzed on 16S rRNA pyrosequencing. Statistical analyses were performed to describe the impact of ivacaftor on the airway microbiota.

## Results and Discussion

### Airway microbial community structure in CF patients

#### 16S rRNA pyrosequencing data

A total 498,373 high-quality reads were generated, with an average 24,918 reads per sample. Based on 97% sequence similarity and after normalization to the lowest number of reads for a sample (14,855 reads), 129 operational taxonomic units (OTUs) were identified, representing 64 genera. Mean sequence length was approximately 420 bp. The UPARSE quality filtering tool ensured high confidence for the identified OTUs [19]. The core microbiota, representing OTUs present in at least 50% of samples [20], comprised only 65 OTUs: i.e., half of all OTUs. Of these 65 OTUs, the 16 accounting for more than 1% of the total number of reads were selected for further analysis (major core microbiota). These 16 OTUs belonged to 11 genera, *Fusobacterium*, *Haemophilus* (specifically, *H*. *parainfluenzae*; *H*. *influenzae* did not belong to the core microbiota), *Prevotella*, *Rothia*, *Staphylococcus* (*S*. *aureus*), *Streptococcus*, *Veillonella*, *Gemella*, *Neisseria*, *Porphyromonas* and *Peptostreptococcus*. These genera were those commonly identified in other studies [10,11,21]. One major difference from previous studies was that *Pseudomonas aeruginosa* was not found to be part of the core microbiota: only 1 OTU assigned to *P*. *aeruginosa* was found in 5 samples obtained from the three patients, but with relative abundance <1% except in 1 sample (S1 Fig); this sample (RM8, S1 Fig), with the highest relative abundance of *P*. *aeruginosa* came from a sample collected after tobramycin treatment had been discontinued, which probably had a significant impact on this particular observation. This low prevalence of *P*. *aeruginosa* can be explained by the young age of the patients, except patient RM who was significantly older (16 years old). This may also explain the wide bacterial diversity within the CF pulmonary core microbiota as compared to findings in adults [6,21]: the present series was pediatric, with a mean age of 12 years, which, according to Cox *et al*., is the age-group with the highest microbial diversity [22]. The study of patients without traditional CF pathogens may be of great interest, as underlined by Zemanick *et al*. [10].

#### Patient-specific airway microbiotas share a common core

**Fig 1:**
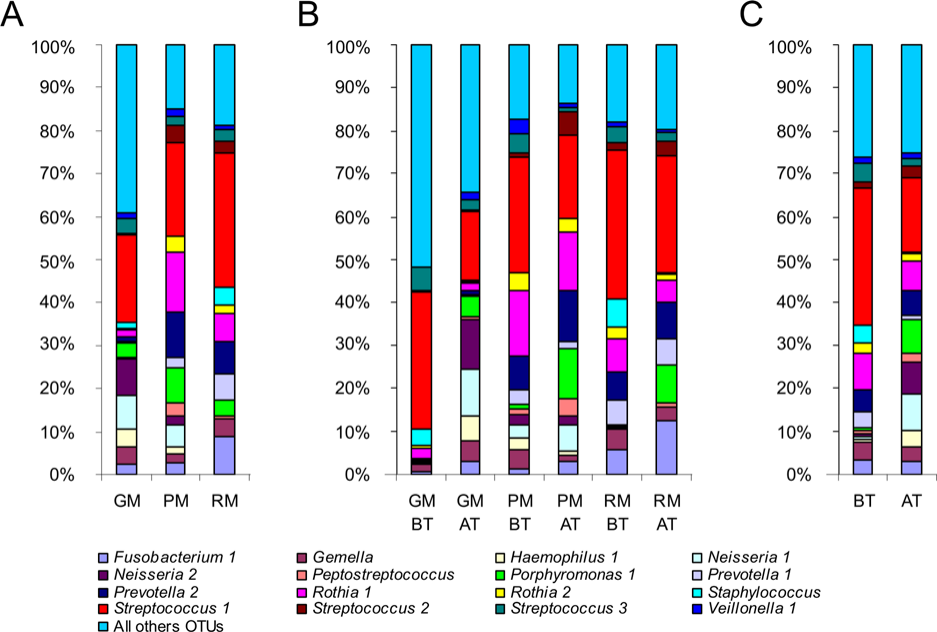
Relative abundance (RA) of OTUs belonging to the major core microbiota. A) RA of OTUs for the three patients (GM, PM, and RM) highlighted that each individual harbored his or her own microbiota, even if several genera were shared. B) RA of OTUs before ivacaftor treatment (BT) and after the beginning of ivacaftor treatment (AT) for each patient. RA of *Streptococcus* 1 showed a tendency to decrease from BT to AT samples, whereas *Porphyromonas* 1 increased. C) Grouping all BT samples (on the left of the graph) and all AT samples (on the right of the graph) confirmed the tendency observed per patient: after ivacaftor treatment, the RA of *Streptococcus* 1 decreased while that of *Porphyromonas* 1 increased.

Each patient had a specific airway microbiota some OTUs being specific to one or two patients (results not shown). However, the 16 OTUs belonging to the core microbiota were shared by all patients, although the variability in their relative abundance clearly highlighted 8 OTUs (Fig 1A). They represented 7 genera (i.e., *Veillonella*, *Streptococcus*, *Rothia*, *Prevotella*, *Porphyromonas*, *Gemella* and *Fusobacterium*) belonging to the endogenous anaerobic microbiota of the oral airway, the composition of which is indistinguishable from the lung microbiota of healthy subjects [3]. Numerous studies have clearly demonstrated that their detection in sputum samples was not the consequence of oral contamination [23,24], which we confirmed by cytological scoring to evaluate salivary contamination. Furthermore, the presence of these bacterial genera suggested that the oral cavity may act as a reservoir for respiratory infection [25,26]. In all patients, *Streptococcus* 1, corresponding to the *S*. *mitis* group, was the most abundant OTU in the core microbiota, in agreement with Maeda’s findings that patients harbored at least 1 viridans streptococcus species, with strong prevalence for the *S*. *mitis* group [27].

**Fig 2:**
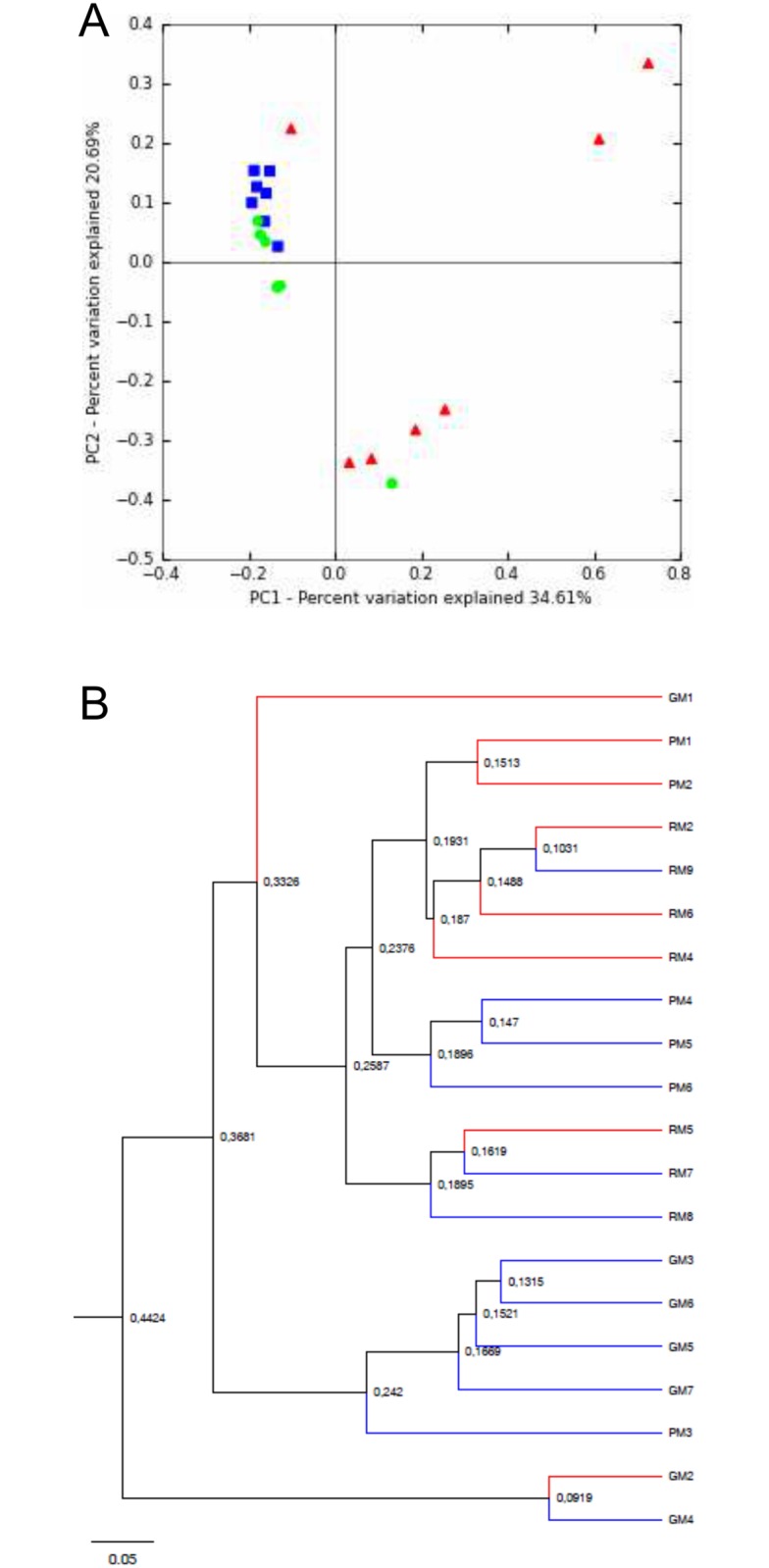
Analysis of microbiota data for the 20 CF sputum samples based on non-phylogenetic distances. A) Principal coordinate analysis of microbial community structure per patient using Bray Curtis distances. PC1 and PC2 represented 55.3% of the variability. Red triangles: patient GM’s samples. Green circles: patient PM’s samples. Blue squares: patient RM’s samples. B) UPGMA clustering of samples using Bray Curtis distances. BT samples are represented by red branches and AT samples by blue branches. The scale bar represents a 5% sequence divergence.

**Fig 3:**
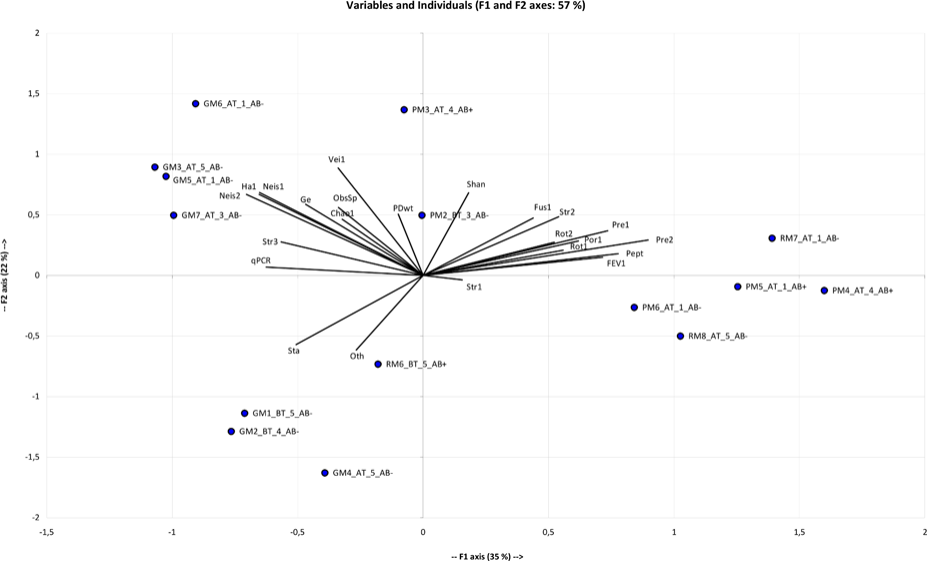
Principal component analysis (PCA) of the 20 sputum samples according to different quantitative variables. For each sample, the name contained the group (BT: before ivacaftor treatment; AT: after the beginning of ivacaftor treatment), cytologic class (1 to 5) and presence (AB+) or absence (AB-) of antibiotic treatment. The F1 and F2 axes explained respectively 35% and 22% of the variability. Only 15 samples are represented, because FEV-1 data were lacking for 5 samples (Table 1). The F1 axis was positively correlated with FEV-1, *Peptostreptococcus* (Pept), *Prevotella* 1&2 (Pre1&2), *Porphyromonas* 1 (Por1), *Rothia* 1&2 (Rot1&2) and *Streptococcus* 2 (Str2). These variables also correlated positively with each other, suggesting that these taxa may be positively correlated with FEV-1 improvement. In contrast, the F1 axis correlated negatively with qPCR, *Haemophilus* 1 (Ha1), *Neisseria* 1&2 (Neis1&2), *Staphylococcus aureus* (Sta) and *Streptococcus* 3 (Str3), indicating that these taxa may be more abundant and with higher bacterial density when respiratory capacity is lower. The F2 axis opposed *S*. *aureus* and OTUs not belonging to the core microbiota (Oth) to *Veillonella* (Veil), *Neisseria* 1&2, *Haemophilus* 1, *Gemella* (Ge) and higher diversity indices (Shannon index (Shan), phylogenetic diversity whole tree (PDwt) and observed species (ObsSp)).

To go further in the comparison of the community structure of samples, principal coordinate analysis (PCoA) and unweighted pair-group method using average linkages (UPGMA) clustering were performed using Bray-Curtis (Fig 2) and UniFrac (S2 Fig) distance metrics. Both analyses highlighted a clustering of samples from patient GM (*Pseudomonas aeruginosa* (*Pa*) status: “never”), whereas the *Pa*-intermittent patients RM and PM exhibited more similar microbiotas (Figs 2 and S2; see Table 1 for patient characteristics). This may suggest that, whereas it was thought that each CF patient harbors a specific airway microbiota [8], shared microbiological history, such as *P*. *aeruginosa* acquisition, can make for common points in the microbiota. Moreover, patient GM was the only one who was not under antibiotherapy at the time of sampling (Table 1), which could also be an explanation. Likewise, principal component analysis (PCA) distinguished GM’s samples, which were all negatively located on the F1 axis (Fig 3).

**Table 1:** Patient characteristics associated with each sputum sample.

Sample name	Date of sputum sampling^a^ (D/M/Y)	Ivacaftor treatment	FEV-1	Antibiotherapy	Antibiotic	Cytological score	Microbiology (cultural method)	*Pa* status [37]
GM1	13 04 12	No	69	No	-	5	Hi, MSSA	Never
GM2	**26 06 12**	No	69	No	-	4	Hi, MSSA	Never
GM3	25 07 12	Yes	94	No	-	5	MSSA, *Sphingomonas parapaucimobilis*, *Pseudomonas fluorescens*	Never
GM4	11 12 12	Yes	94	No	-	5	Hi, MSSA, *Branhamella catarrhalis*	Never
GM5	07 03 13	Yes	92	No	-	1	MSSA	Never
GM6	27 06 13	Yes	91	No	-	1	MSSA, *P*. *fluorescens*	Never
GM7	03 10 13	Yes	83	No	-	3	Hi, MSSA	Never
PM1	26 07 12	No	NA	Yes	Colimycin aerosol	4	No pathogenic germs	Intermittent
PM2	**03 10 12**	No	84	No	-	3	No pathogenic germs	Intermittent
PM3	23 01 13	Yes	117	Yes	Tobramycin nebulization	4	No pathogenic germs	Intermittent
PM4	20 02 13	Yes	104	Yes	Tobramycin nebulization	4	MSSA, *Pa*	Intermittent
PM5	22 05 13	Yes	100	Yes	Tobramycin nebulization	1	MSSA	Intermittent
PM6	22 08 13	Yes	98	No	-	1	MSSA	Intermittent
RM2	13 02 13	No	NA	No	-	1	MSSA, *Pa*	Intermitent
RM4	22 04 13	No	NA	No	-	5	MSSA	Intermittent
RM5	01 06 13	No	NA	No	-	5	MSSA	Intermittent
RM6	**11 07 13**	No	84	Yes	Tobramycin nebulization	5	MSSA	Intermitent
RM7	07 08 13	Yes	103	No	-	1	No pathogenic germs	Intermittent
RM8	26 09 13	Yes	108	No	-	5	MSSA, *Pa*	Intermitent
RM9	28 10 13	Yes	NA	Yes	Colimycin aerosol	1	No pathogenic germs	Intermittent

^a^Dates in bold correspond to the beginning of ivacaftor treatment; sputum samples corresponding to the first day of ivacaftor treatment were collected before its administration.

FEV-1: forced expiratory volume in 1 second.

Hi: *Haemophilus influenzae*, MSSA: methicillin sensitive *Staphylococcus aureus*, *Pa*: *Pseudomonas aeruginosa*.

### CF airway microbiota dynamics throughout ivacaftor treatment

#### Study design for optimal assessment of microbiota dynamics, and its limitations

For each CF patient, between 6 and 7 sputum samples were analyzed over a period of 13 months covering 2 periods: before (BT) and after (AT) ivacaftor treatment initiation (Table 1). Sampling every 2 months on average with an average 3 samples per period enabled better assessment of baseline and post-treatment microbiotas. This is a major difference compared to the only other study on the subject, in which only 2 samples per patient were analyzed [14]. One limitation of the present study is that it would have been better to have had more evenly spaced samples. For robustness, statistical analyses were conducted pooling all BT and AT sequencing data in order to compare them. AT follow-up data covered both short- and long-term periods, with a lag-time of 1 to 16 months after initiation of ivacaftor. Phase-3 studies showed that the clinical benefit of ivacaftor can be seen within 15 days, and are maintained with treatment [15,16]. Results obtained for these three patients were consistent with those of clinical trials: FEV-1 improved systematically after ivacaftor administration (Table 1). Furthermore, this close follow-up was necessary considering the young age of the patients and the likely complexity of their airway microbiota [22]. The main limitation of the study was the small number of patients: we sought to focus on patients in the same age range and attending the same CF center in order to limit confounding factors: but patients with the G551D mutation are rare as this mutation accounts for only 4% of CFTR alleles. Moreover, ivacaftor is not presently indicated for patients under 6 years of age, which further restricted candidates for the study. Another limitation of the study was the multiplicity of antibiotherapy schedules received by the 3 patients. Patient GM was receiving no antibiotics as the time of sampling time; patient PM was receiving antibiotics by nebulization at almost each time of sampling; and patient RM occasionally received inhaled antibiotics (Table 1). This may have led to interactions, disrupting the effects of ivacaftor on the microbiota. Although studies tend to show that, in the long term, the airway microbiota is resilient [25], it would be very interesting to apply the approach of Zhao *et al*., who recently addressed the challenging question of the impact of antibiotic exposure on the microbiota by testing different models [28]. Other variables such as age and gender [28] may also act on diversity. Therefore, it will be important to conduct larger studies addressing this issue and taking account of a huge number of variables in order to implement an optimally reliable model of the relationship between disease and treatment.

#### Overall stability of bacterial density

The evolution of total bacterial load was measured by quantitative PCR (qPCR). The DICO internal control (Argène) showed similar DNA extraction efficiency in each sample and the absence of PCR inhibitors. The mean quantity of 16S rRNA gene copies/mL was 8.9 log10 (standard deviation: 0.54), with no significant difference between the BT and AT periods (Colin White test, p>0.05; S2 Table). These data suggest that ivacaftor treatment did not disturb total bacterial density, in agreement with Rowe [14]. CF microbiota stability seems perennial, despite any factors liable to disturb it, as previously described for different clinical states [11,25,29]. It might have been expected that the ivacaftor-related improvement in mucociliary clearance [14] would have at least presaged a significant decrease in microbial density. One possible explanation often discussed [10] is that increased contamination by oral bacterial species may counterbalance the reduction in lung species. The issue of contamination requires careful consideration [30]. Obtaining samples from the lower airways involves passage through regions that are typically heavily colonized by microbes [31], with a risk of comparing microbiota samples from different niches. To take this phenomenon into account, we applied a quality score to evaluate the degree of salivary contamination (Table 1), and we were able to demonstrate that there was no significant impact of salivary contamination, whether on microbial composition (S3 Fig) or the other variables analyzed (S2 Table), as the 4 observed cytological scores (Table 1) were scattered on the PCA graph (Fig 3).

#### Greater dissimilarity between microbial communities after ivacaftor administration

**Fig 4:**
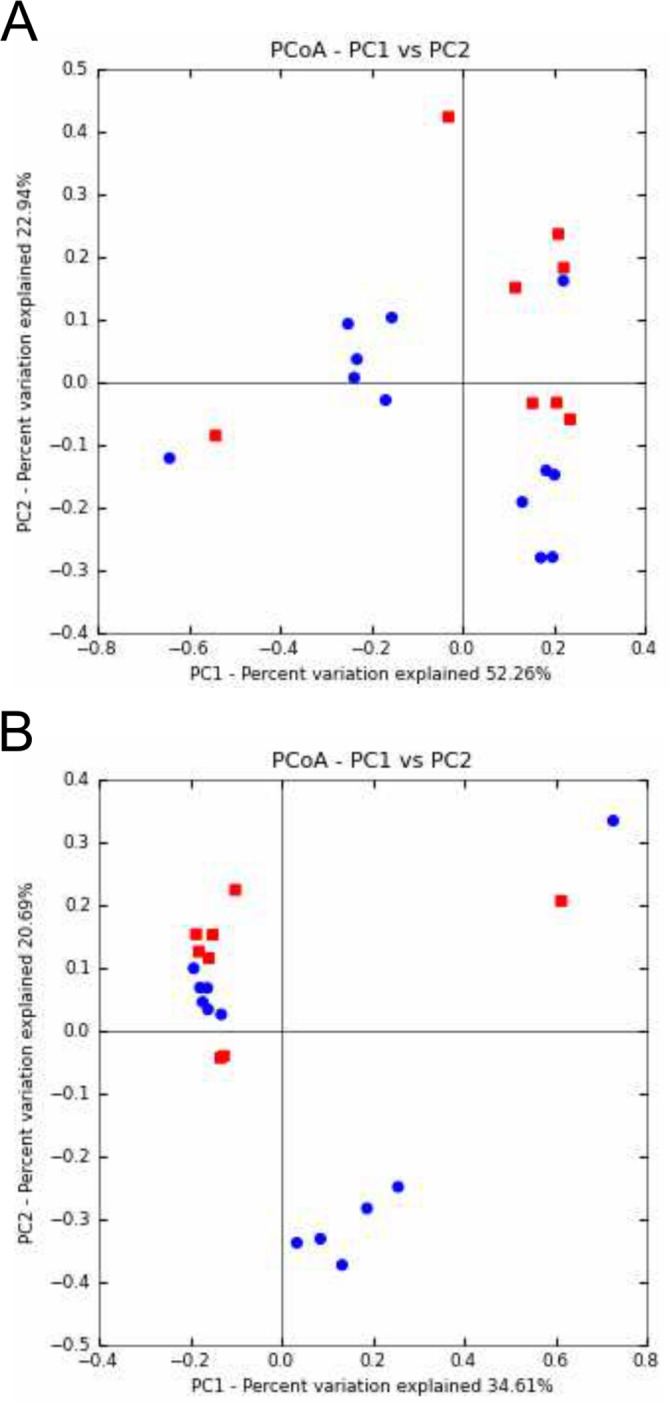
Principal coordinates analysis (PCoA) of CF sputum samples according to ivacaftor treatment and microbial community composition and abundance. A) PCoA of microbial community structures using weighted and normalized UniFrac phylogenetic distances. A clustering of 6 of the 8 BT samples (before ivacaftor treatment; red squares) was observed. Conversely, AT samples (after ivacaftor treatment; blue circles) appeared scattered on the graph. PC1 and PC2 represented 75.2% of the variability. B) PCoA of microbial community structures using Bray Curtis non-phylogenetic distances. Seven of the 8 BT samples (red squares) were clustered. PC1 and PC2 represented 55.3% of the variability.

PCoA based on UniFrac and Bray Curtis distance metrics revealed groupings of respectively 6 and 7 out of the 8 BT samples (Fig 4A and 4B). Conversely, the AT samples did not cluster, suggesting broader dissimilarity in microbial community composition and relative abundance after ivacaftor treatment. These two observations, taken together with a tendency for greater diversity in AT samples (S4 Fig), suggest that ivacaftor may disrupt the CF microbiota, even if no significant differences were observed on the Colin White test (S2 Table). This tendency corroborates the hypothesis made by Rowe *et al*., who suggested increased microbial diversity resulting from ivacaftor administration [14]. As for other clinical situations in CF [11,25,32], increased biodiversity may be supposed to be associated with improved respiratory function, as well as other clinical endpoints in phase-3 studies of ivacaftor [15,16].

#### Two components of the airway microbiota showed common evolution under ivacaftor treatment

Although the dynamics of most OTUs were patient-specific (Fig 1B), analyses pooling the common core microbiotas of BT and AT samples (compared using Colin White test) revealed 2 OTUs with significant evolution (Fig 1C, S2 Table). The relative abundance of *Streptococcus* 1 (*S*. *mitis* group) decreased after ivacaftor treatment initiation (p<0.05), while that of *Porphyromonas* 1 increased in all 3 patients (p<0.01); these significant differences were the only ones observed between the BT and AT groups (S2 Table). One possible explanation of the two-way dynamics of the airway microbiota (*Streptococcus* 1 decrease concomitant to *Porphyromonas* 1 increase) is in terms of the antibacterial properties of ivacaftor recently described by Reznikov *et al*. [18]: ivacaftor was shown to be specifically active on Gram-positive bacteria; the fact that *Porphyromonas* is Gram-negative, unlike *Streptococcus*, may explain these results in part. The authors suggested that ivacaftor’s structure (containing a quinolone ring) could enable more selectivity against Gram-positive versus Gram-negative bacteria [18]. A second explanation could concern inter-species relations within bacterial communities. The two bacteria in question were previously shown to be negatively correlated in dental plaque [33]: *Streptococcus mitis* enhanced the capacity to limit *P*. *gingivalis* and *P*. *intermedia* adhesion [34]. By analogy, the diminution of the *S*. *mitis* population due to ivacaftor’s antibiotic effect may have allowed *Porphyromonas* 1 to adhere better and then develop.

#### Potential involvement of several anaerobes in respiratory capacity improvement

Zemanick *et al*. demonstrated that anaerobes are associated with less inflammation and better lung function [10]. We confirmed this finding. In the PCA graph (Fig 3), the F1 axis was positively correlated with FEV-1 and with 8 OTUs. Two of these 8 OTUs were significantly positively correlated with FEV-1, as highlighted by the Spearman correlation test (Table 2): *Prevotella* 2 (r = 0.73, p<0.05) and *Porphyromonas* 1 (r = 0.62, p<0.1). Some showed correlation on the adjusted Spearman correlation test (Table 3): *Prevotella* 2 was significantly positively correlated with *Peptostreptococcus* (r = 0.69, p<0.05) and *Prevotella* 1 (r = 0.61, p<0.05); *Porphyromonas* 1 was significantly positively correlated with *Peptostreptococcus* (r = 0.68, p<0.05); and *Prevotella 1* was significantly positively correlated with *Streptococcus* 2 (r = 0.65, p<0.05).

**Table 2:** Association between relative abundance of OTUs belonging to the major core microbiota and FEV-1 (n = 15 sputum samples).

OTUs of the core microbiota and 16S rRNA qPCR	FEV-1		
	SCC	p-value	FDR adjusted p-value
*Gemella*	-0.453	0.0899	0.1856
*Haemophilus* 1	-0.2586	0.3521	0.5282
*Staphylococcus*	**-0.5197**	**0.0471**	0.1494
*Prevotella* 1	0.4566	0.0871	0.1856
*Streptococcus* 1 (*S*. *mitis* group)	-0.2363	0.3964	0.5489
*Peptostreptococcus*	**0.5143**	**0.0498**	0.1494
*Streptococcus* 2 (*S*. *salivarius* group)	**0.5246**	**0.0447**	0.1494
*Neisseria* 1	-0.1766	0.5289	0.6347
*Fusobacterium* 1	0.4494	0.0928	0.1856
*Rothia* 1	0.0949	0.7366	0.7799
*Veillonella* 1	-0.0967	0.7318	0.7799
***Porphyromonas* 1**	**0.6231**	**0.0131**	**0.0786**
*Neisseria* 2	-0.3652	0.1807	0.2957
***Prevotella* 2**	**0.7305**	**0.002**	**0.0360**
*Rothia* 2	0.0555	0.8442	0.8442
***Streptococcus* 3 (*S*. *anginosus* group)**	**-0.6822**	**0.0051**	**0.0459**
All others OTUs	-0.2077	0.4576	0.5883
qPCR	-0.4136	0.1254	0.2257

SCC: Spearman's correlation coefficient (r).

FDR: false discovery rate.

Significant correlations after FDR adjustment are in bold (threshold = 0.1).

**Table 3:** FDR adjusted p-values associated with Spearman correlations between relative abundance (RA) of OTUs belonging to the major core microbiota and qPCR results.

	Ge	Ha1	Sta	Pre1	Str1	Pept	Str2	Neis1	Fus1	Rot1	Vei1	Por1	Neis2	Pre2	Rot2	Str3	Oth	qPCR
Ge	/	0.1799	0.8597	0.5909	0.5534	0.7647	0.7397	0.2266	0.8099	0.9092	**0.0150**	0.4285	0.1799	0.5534	0.8597	0.1431	0.7318	0.8331
Ha1	0.1799	/	0.4285	0.1897	0.1897	0.7397	0.5534	**2.10–7**	0.9840	0.2236	**0.0154**	0.7324	**3.10–6**	0.2576	0.2576	0.5909	0.7766	0.0254
Sta	0.8597	0.4285	/	0.9045	0.6296	0.0817*	0.5879	0.5534	0.3932	0.2494	0.2638	0.1799	0.7937	0.3114	0.3339	0.8199	0.1431	0.7101
Pre1	0.5909	0.1897	0.9045	/	0.3339	0.5073	**0.0214**	0.2247	0.4345	0.1192	0.5474	0.8815	0.3344	**0.0422**	0.0637*	0.9092	0.6289	**0.0030**
Str1	0.5534	0.1897	0.6296	0.3339	/	0.7937	0.4285	0.1799	0.7833	0.2473	0.8597	0.1896	0.2159	0.7397	0.2494	0.0817*	0.4876	0.2169
Pept	0.7647	0.7397	0.0817*	0.5073	0.7937	/	0.7600	0.5117	0.1919	0.1824	0.8199	**0.0163**	0.4222	**0.0160**	0.2638	0.1799	0.7101	0.8244
Str2	0.7397	0.5534	0.5879	**0.0214**	0.4285	0.7600	/	0.5909	0.1325	0.4676	0.5474	0.7949	0.4285	0.1439	0.4345	0.9840	0.2976	**0.0163**
Neis1	0.2266	**2.10–7**	0.5534	0.2247	0.1799	0.5117	0.5909	/	0.7600	0.2576	**0.0163**	0.7600	**5.10–8**	0.1799	0.3344	0.7600	0.8151	0.0687*
Fus1	0.8099	0.9840	0.3932	0.4345	0.7833	0.1919	0.1325	0.7600	/	0.7600	0.9092	0.2159	0.7334	0.2473	0.7600	0.1833	0.5534	0.2860
Rot1	0.9092	0.2236	0.2494	0.1192	0.2473	0.1824	0.4676	0.2576	0.7600	/	0.8151	0.8815	0.2638	0.1327	**1.10–10**	0.5276	0.1799	0.4529
Vei1	**0.0150**	**0.0154**	0.2638	0.5474	0.8597	0.8199	0.5474	**0.0163**	0.9092	0.8151	/	0.9045	**0.0197**	0.8286	0.7600	0.1964	0.2748	0.5474
Por1	0.4285	0.7324	0.1799	0.8815	0.1896	**0.0163**	0.7949	0.7600	0.2159	0.8815	0.9045	/	0.8099	0.2594	0.8199	0.0637*	0.7318	0.7061
Neis2	0.1799	**3.10–6**	0.7937	0.3344	0.2159	0.4222	0.4285	**5.10–8**	0.7334	0.2638	**0.0197**	0.8099	/	0.2218	0.4285	0.5792	0.9045	0.1254
Pre2	0.5534	0.2576	0.3114	**0.0422**	0.7397	**0.0160**	0.1439	0.1799	0.2473	0.1327	0.8286	0.2594	0.2218	/	0.1431	0.4489	0.5769	0.0817*
Rot2	0.8597	0.2576	0.3339	0.0637*	0.2494	0.2638	0.4345	0.3344	0.7600	**1.10–10**	0.7600	0.8199	0.4285	0.1431	/	0.5276	0.1431	0.3498
Str3	0.1431	0.5909	0.8199	0.9092	0.0817*	0.1799	0.9840	0.7600	0.1833	0.5276	0.1964	0.0637*	0.5792	0.4489	0.5276	/	0.4529	0.6445
Other	0.7318	0.7766	0.1431	0.6289	0.4876	0.7101	0.2976	0.8151	0.5534	0.1799	0.2748	0.7318	0.9045	0.5769	0.1431	0.4529	/	0.8286
qPCR	0.8331	**0.0254**	0.7101	**0.0030**	0.2169	0.8244	**0.0163**	0.0687*	0.2860	0.4529	0.5474	0.7061	0.1254	0.0817*	0.3498	0.6445	0.8286	/

Significant correlations after FDR adjustment are represented in bold (threshold: 0.05), or are indicated by a star (threshold: 0.1). Negative correlations are underlined (S3 Table).

Ge: *Gemella*, Str2: *Streptococcus* 2 (*S*. *salivarius* group), Neis2: *Neisseria*, Ha1: *Haemophilus* 1, Neis1: *Neisseria* 1, Pre2: *Prevotella* 2, Sta: *Staphylococcus aureus*, Fus1: *Fusobacterium* 1, Rot2: *Rothia* 2, Pre1: *Prevotella* 1, Rot1: *Rothia* 1, Str3: *Streptococcus* 3 (*S*. *anginosus* group), Str1: *Streptococcus* 1 (*S*. *mitis* group), Vei1: *Veillonella* 1, Other: OTUs out of the major core microbiota, Pept: *Peptostreptococcus*, Por1: *Pophyromonas* 1.

### Potential implication of Streptococcus 3 (*S*. *anginosus* group) in respiratory impairment (Fig 5)

PCA (Fig 3) revealed that the F1 axis was negatively correlated with 5 OTUs, some of which (*Gemella*, *Haemophilus* 1, *Neisseria* 1&2, *Veillonella* 1) were themselves positively correlated as shown by the adjusted Spearman correlation test (Table 3). The F1 axis was also negatively correlated with *Streptococcus* 3 (*S*. *anginosus* group). *Streptococcus* 3 was significantly negatively correlated with FEV-1 (r = -0.68, p<0.05; Fig 3, Table 2) and with *Porphyromonas* 1 (r = -0.58, p<0.1; Table 3), an anaerobe significantly positively correlated with FEV-1.

**Fig 5:**
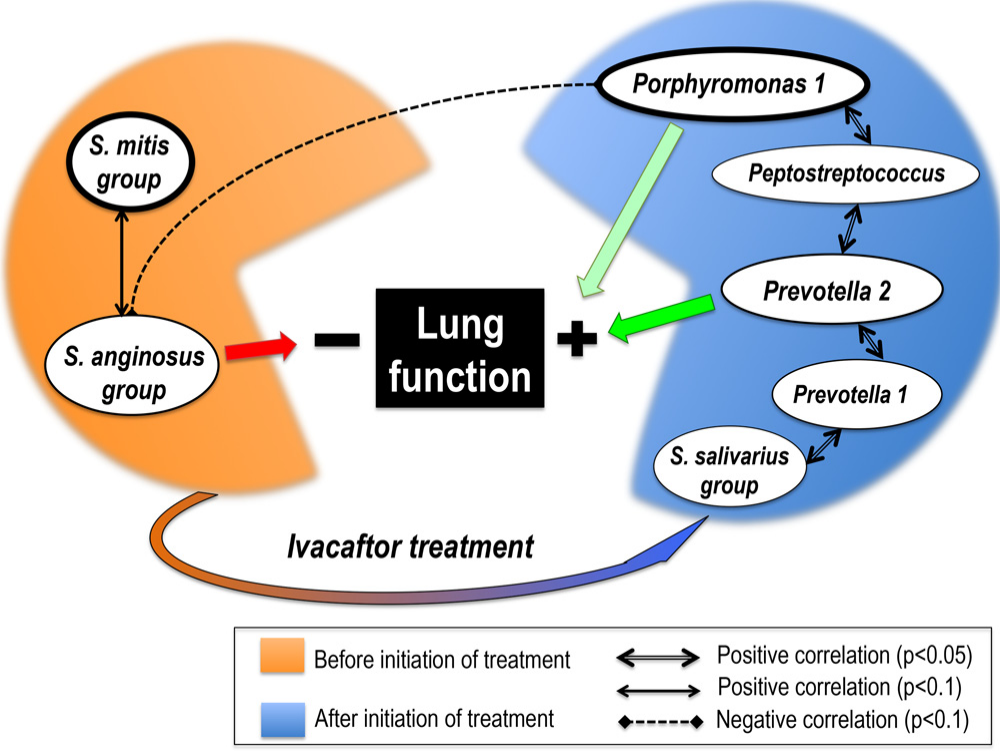
Dynamics and interrelations of 7 key-role OTUs throughout ivacaftor treatment, and their correlations with lung function. *Streptococcus* 1 (*S*. *mitis* group) and *Porphyromonas* 1 were the two OTUs for which a significant association with ivacaftor treatment period emerged (Colin-White test; See S2 Table): *Streptococcus* 1 (*S*. *mitis* group) was associated with sputum samples collected before (p<0.05) and *Porphyromonas* 1 with samples collected after initiation of treatment (p<0.01). The depicted correlations between OTUs were all statistically significant with the adjusted p-values (Spearman correlation test; see Table 3). Significant correlations between OTUs and lung function (on FEV-1 test) are shown by red arrow when negative (p<0.05) and green arrow (light green if p<0.1; dark green if p<0.05) when positive (adjusted p-values; see Table 2).

The role of viridans streptococci remains under debate, with conflicting or contradictory correlations reported: either poor outcome or stability [9,35]. Our results highlighted these oppositions between *Streptococcus* species. Although *Streptococcus* 1 (*S*. *mitis* group) did not significantly correlate with FEV-1 (Fig 3, Table 2), the Colin White test showed a significantly lower relative abundance in the AT group, which showed higher FEV-1 values. Moreover, *Streptococcus* 1 showed a positive correlation with *Streptococcus* 3 (r = 0.56, p<0.1; Table 3), an OTU significantly negatively correlated with FEV-1 (r = -0.62, p<0.05, Table 2), and negatively correlated with *Porphyromonas* 1 (r = -0.58, p<0.1, Table 3). Conversely, *Streptococcus* 2 (*S*. *salivarius* group) was significantly positively correlated with *Prevotella* 1 (r = 0.65, p<0.05), an anaerobe potentially positively associated with FEV-1 (Fig 5). The difference between the 3 groups of streptococci in terms of correlation with respiratory function highlights the need for streptococci to be the focus of dedicated study, with accurate species identification.

To sum up, two congruent statistical analyses, the Colin White and Spearman correlation tests, highlighted an association between higher relative abundance of *Streptococcus* 1&3, BT group and lower FEV-1 values, in contrast to an association between higher relative abundance of *Peptostreptococcus*, *Prevotella* 2, *Porphyromonas* 1, *Prevotella* 1, and *Streptococcus* 2 (*S*. *salivarius* group), AT group, and higher FEV-1 values (Fig 5). These 7 OTUs could represent potential new biomarkers of either deleterious or healthy airway microbiota, interesting for the follow-up of respiratory capacity under ivacaftor. It may be hypothesized that the potentially beneficial bacteria selected by ivacaftor may contribute to improved respiratory function in G551D patients. However, this promising hypothesis remains to be confirmed as it is very difficult to assess the direct impact that ivacaftor itself has on the airway microbiota, given the varied courses of antibiotics that two of the patients received during the study period; therefore, a larger study has to be performed on more patients and samples, with stratification according to antibiotic courses.

Using an *in-vitro* model mimicking the host and its microenvironment would also be relevant, as recently proposed by Crabbé *et al*. [36].

## Conclusion

Ivacaftor is a very promising first-in-class molecule, providing improvement in the clinical (including infectious) parameters of CF patients [14], and does not induce major changes in CF airway microbiota density and composition, but seems to enhance bacterial diversity. Its antibiotic properties could explain the significant decrease in the relative abundance of *Streptococcus* 1 (*S*. *mitis* group) counterbalanced by an increase in the relative abundance of *Porphyromonas* 1. It would be interesting to confirm these opposing microbial dynamics (with a possible beneficial side-effect on lung function) in a larger number of patients, and to monitor immune response. It would also be interesting to decipher the mechanisms involved in the evolution of these bacterial populations, which represent potential biomarkers of lung function. Given the effect of ivacaftor on the intestinal fluid, studying the gut microbiota would be of interest too. Finally, as clinical trials have focused on patients with more prevalent mutations (e.g., dF508 mutation), it is becoming obvious that, with the advent of new treatments that will soon concern almost all CF patients, microbiota analysis offers potentially valuable biomarkers for follow-up, and a means of improving our understanding of the pathophysiology of the disease.

## Methods

### Ethics statements, patients, data collection, and sputum sampling

The local institutional review board (*Comité de Protection des Personnes Ouest VI*) approved the protocol. All patients and relatives gave written informed consent. The specimen archive was registered with the French Ministry of Research and the regional hospital admissions agency (*Agence Régionale de l’Hospitalisation)* under the number #DC-2008-214.

Three CF children (GM, PM and RM), all female, aged 10, 11 and 16 years respectively, attending the Roscoff (France) CF Center were included in the study between April 2012 and October 2013. Sputum sampling was performed at a mean 3 months before initiation of ivacaftor and every 2 months (68.9 days) on average for a mean 10 months during treatment. Seven sputum specimens were collected for patients GM and RM and 6 for patient PM. The data collected for these 20 sputum samples included pulmonary function, antibiotherapy and *P*. *aeruginosa* status according to Lee’s definition [37] (Table 1).

### Sputum processing

Sputum samples were processed using a standard operating procedure [38]. As recommended by the French guidelines [38], sputum sample quality was verified by cytological examination of fresh smears under a ×10 lens microscope (×100 magnification) and classified according to the number of epithelial cells (EC) and leukocytes (L) (class 1: >25 EC, <10 L in a given microscopic field; class 2: >25 EC, 10–25 L; class 3: >25 EC, >25 L; class 4: 10–25 EC, >25 L; class 5: <10 EC, >25 L). Class 1 and 2 samples, highly contaminated by saliva, were classified as poor quality; class 3 and 4 samples were classified as moderate quality, and class 5 as appropriate quality. Each sputum sample was mixed with an equal volume of dithiothreitol (Digest-EUR Eurobio, Courtaboeuf, France) and incubated at room temperature for 30 min. Ten μl liquefied sputum, pure or diluted 1/1,000, was inoculated and incubated in several non-selective and selective media, and conventional microbiological diagnosis was conducted as previously described [39]. After processing for bacterial culture, the liquefied sputum samples were stored at -80°C for further analysis.

### DNA extraction

DNA extraction was performed as previously described [39] with the QIamp DNA mini kit (QIAGEN, USA) according to the manufacturer’s guidelines. One hundred and fifty microliters of each sputum sample were loaded into new tubes and sonicated for 5 min (Branson 200, USA). Then, proteinase K (0.8 mg per sample, QIAGEN, USA), ATL buffer (180 μl per sample) and the universal control IC2 (10μl per sample) from the DICO Extra r-gene kit (Argène, Verniolle, France) were added, followed by incubation for 3h at 56°C with 15 s vortexing every 30 min. Purified DNA was harvested with elution buffer and quantified using a NanoVue Plus spectrophotometer (GE Healthcare, USA).

### Quantitative PCR

Primers, probe and methods previously described by Zemanick *et al*. were used to quantify total bacterial population by qPCR targeting 16S rRNA gene [10]. qPCR was performed using the TaqMan Gene Expression Master Mix (Applied Biosystems, USA) and the AB7500 Fast Real-Time PCR system (Applied Biosystems, USA). For the standard curve, the *Pseudomonas aeruginosa* PAO1 strain was used. Based on its genome size (6,264,404 bp) and its 16S rRNA copy number (4 copies), DNA concentration was converted into 16S rRNA copy number. Thus, the standard curve obtained (R² = 0.998, slope = -3.974) determined one 16S rRNA copy number per sample, according to the cycle threshold value obtained. The IC2 internal control used the premix included in the DICO extra r-gene kit (Argène) with the Hotstart enzyme (Qiagen).

### Sample preparation for pyrosequencing

All DNA samples were diluted to 50 ng/μl and a ~420 pb V3–V4 hypervariable region of the 16S rRNA gene was amplified in duplicate for each sample (total reaction volume, 50 μl) using primers 347F and 803R [40]. Primers comprised an adaptor sequence (A for the reverse primer, B for the forward primer, Life Technologies), and each reverse primer had its own multiplex identifier sequence. After pooling amplification products and checking that approximately the same amount of amplicons was produced for each sample by electrophoresis, samples were assayed by Agilent Bioanalyzer 2100 (Agilent Technologies). The same amounts were pooled and sequenced by GATC-Biotech (Konstanz, Germany) on a 454 FLX-titanium sequencer (Roche). The DNA sequencing data was deposited in the National Center for Biotechnology Information (NCBI) Short Read Archive database under the BioProject number PRJNA258369 following NCBI guidelines [41].

### Bioinformatics tools and statistical analysis

The 670,843 raw reads obtained after pyrosequencing were processed as described in the UPARSE pipeline (http://drive5.com/usearch/manual/uparse_cmds.html) with the following quality-filtering parameters: truncation length, 250 bp; truncation to the first nucleotide with quality score <20; maximal expected error, 0.25 [19]. After removal of singletons, sequences were clustered into OTUs based on a sequence similarity level of 0.97 using the UPARSE-OTU algorithm. Then, chimeric sequences were checked using UCHIME [7] against the GOLD database. QIIME [42] was used to normalize the OTU table to the smallest number of reads (14,855) obtained in a sample. The Greengenes database (May 2013 release: gg_13_5_99) was used for taxonomic assignation (http://greengenes.lbl.gov) [43] based on the BLAST algorithm. Taxonomic assignments were also checked on the RDP (http://rdp.cme.msu.edu) [44], SILVA (http://www.arb-silva.de/aligner/) [45] and NCBI databases (http://blast.ncbi.nlm.nih.gov). Ecological indices were calculated using QIIME. More precisely, phylogeny and non-phylogeny based metrics were used to describe alpha (phylogenetic diversity whole tree, observed species, Chao1 and Shannon index), and beta diversity (UniFrac and Bray Curtis).

Samples obtained before initiation of ivacaftor treatment were referred to as BT (Before Treatment), and those obtained after as AT (After Treatment). Statistical analysis used StatBox software (Grimmersoft, Paris, France), and R software (Hmisc and *stats* packages; http://www.r-project.org). Conventional checks (normality test, homogeneity of variance) were made before applying the appropriate statistical test. The non-parametric Colin White test [46] was performed on Excel software.

Given that ivacaftor treatment improves FEV-1 [14–16], we checked for a potential correlation between FEV-1 and OTUs belonging to the core microbiota on all samples. Thus, the association between the quantitative variable FEV1 and the relative abundance of OTUs was tested by Spearman correlation (Table 2; n = 15). The same test was used to check the correlation between OTUs belonging to the major core microbiota (S3 Table; n = 20). The p-values associated with the correlation coefficients (Table 2; S4 Table) were adjusted for false discovery using the Benjamini-Hochberg procedure [47] on the R *stats* package (Table 3). To be able to summarize all data in a graph and analyze links between FEV-1 values, qPCR data and relative abundance of OTUs belonging to the major core microbiota, principal component analysis (PCA) was performed. Each sample was named according to BT versus AT status, cytologic class (1 to 5) and presence (AB+) or absence (AB-) of antibiotic treatment.

## Supporting Information

S1 Text (DOCX)

S1 Fig Representation of the relative abundance of OTUs not belonging to the major core microbiota for each of the 20 samples. (TIF)

S2 Fig Analysis of microbiota data for the 20 CF sputum samples based on phylogenetic distances. (TIF)

S3 Fig Relative abundance (RA) of OTUs belonging to the major core microbiota for all samples (n = 20). The very high RA of “All other OTUs” in samples GM2 and GM4 can be explained by the high prevalence of *Haemophilus* 2 (*Haemophilus influenzae*) in these samples (S1 Fig).
(TIF)

S4 Fig Alpha diversity metrics of CF airway microbiota according to ivacaftor treatment or not. (TIF)

S1 Table Coordinates and Cosine square (Cos^2^) of variables on F1 and F2 axes. (PDF)

S2 Table Quantitative variables used for statistical analysis and p-values on Colin White test for all samples, comparing the BT (before treatment) and AT (after treatment) groups for relative abundance of OTUs. (PDF)

S3 Table Correlation (Spearman test) between relative abundance of OTUs belonging to the major core microbiota and qPCR results. (PDF)

S4 Table Uncorrected p-values associated with Spearman correlations between relative abundance of OTUs belonging to the major core microbiota and qPCR results. (PDF)
