# Supplementary material for: Impact of the CFTR-Potentiator Ivacaftor on Airway Microbiota in Cystic Fibrosis Patients Carrying A G551D Mutation
Source: PLoS One. 2015 Apr 8;10(4):e0124124. doi: 10.1371/journal.pone.0124124 (PMC4390299; doi:10.1371/journal.pone.0124124)
Supplement: S1 Text — (DOCX) [file pone.0124124.s001.docx]

**Impact of the CFTR-potentiator ivacaftor on airway microbiota**

**in cystic fibrosis patients carrying a G551D mutation**

Cédric Bernarde, Marlène Keravec, Jérôme Mounier, Stéphanie Gouriou, Gilles Rault, Claude Férec, Georges Barbier, Geneviève Héry-Arnaud

**Results and Discussion**

**Contradictory taxonomic assignations**

With sequence alignment on different databases (Greengenes, RDP, SILVA, NCBI), *Streptococcus* 1 was identified as *S. mitis* and *S. parasanguinis* with the best score on RDP (1.0), and as *S. parasanguinis* only on NCBI (coverage=100%, identity=100%). It was concluded that *Streptococcus* 1 corresponded to the *S. mitis* group, associated with the oral cavity but also identified in lung [1-2]. Similarly, *Streptococcus* 2 and *Streptococcus* 3 were assigned to the *S. salivarius* and *S. anginosus* group, respectively. *Porphyromonas catoniae* was suggested by RDP, but with a too low score (0.801) and identity percentage on NCBI (96%) to confirm *Porphyromonas* 1 species identity.

**Variability observed on PCA was mainly driven by FEV-1**

PCA highlighted interesting correlations (Fig. 3). The F1 axis revealed an opposition between 5 AT samples (RM7-8, PM4-5-6) sharing a mean FEV-1 value of 102.6%, and 4 AT samples (GM3-5-6-7) sharing a mean FEV-1 value of 90% (Table 1). The opposition between these 2 groups seemed to be associated with FEV-1, which was positively correlated with the F1 axis. Thus, AT respiratory capacity had quite a strong influence on sample differentiation. However, it did not affect the strong associations between higher FEV-1 values and AT group and lower FEV-1 values and BT group (Table 1, [3-5]). It is important to take into account that each patient had his or her individual respiratory capacity, and patient GM clearly had the lowest respiratory capacity (Table 1): patient GM’s highest FEV-1 values were not far from the lowest values of patients PM and RM.

**The development of certain bacterial populations may be associated with bacterial density**

The qPCR variable was negatively correlated with FEV-1 but not significantly (r=-0.41, p=0.2257, Table 2), and previous studies showed that bacterial density was lower in healthy than in CF lungs [3]. Five OTUs correlated significantly with qPCR, 3 negatively (*Prevotella* 1&2, *Streptococcus* 2), and 2 positively (*Haemophilus* 1, *Neisseria* 1) (Table 3). Thus, the development of the *Prevotella* 1&2 and *Streptococcus* 2 populations seemed to be enhanced by lower bacterial density, as if they were more sensitive to territorial competition. Conversely, *Haemophilus* 1 and *Neisseria* 1 may have the capacity to develop despite higher bacterial density, which could be directly associated with their own development.

**References**

1. Maeda Y, Elborn JS, Parkins MD, Reihill J, Goldsmith CE, Coulter WA, et al. Population structure and characterization of viridans group streptococci (VGS) including *Streptococcus pneumoniae* isolated from adult patients with cystic fibrosis (CF). J Cyst Fibros. 2011;10:133–139.

2. Nakajima T, Nakanishi S, Mason C, Montgomery J, Leggett P, Matsuda M, et al. Population structure and characterization of viridans group streptococci (VGS) isolated from the upper respiratory tract of patients in the community. Ulster Med J. 2013;82:164–168.

3. Rowe SM, Heltshe SL, Gonska T, Donaldson SH, Borowitz D, Gelfond D, et al. Clinical Mechanism of the CFTR Potentiator Ivacaftor in G551D-Mediated Cystic Fibrosis. Am J Respir Crit Care Med. 2014;90:175-84.

4. Ramsey BW, Davies J, McElvaney NG, Tullis E, Bell SC, Dřevínek P, et al. A CFTR potentiator in patients with cystic fibrosis and the G551D mutation. N Engl J Med. 2011;365:1663–1672.

5. Davies JC, Wainwright CE, Canny GJ, Chilvers MA, Howenstine MS, Munck A, et al. Efficacy and safety of ivacaftor in patients aged 6 to 11 years with cystic fibrosis with a G551D mutation. Am J Respir Crit Care Med. 2013;187:1219–1225.
