# Supplementary figures and images for: Impact of the CFTR-Potentiator Ivacaftor on Airway Microbiota in Cystic Fibrosis Patients Carrying A G551D Mutation
Source: PLoS One. 2015 Apr 8;10(4):e0124124. doi: 10.1371/journal.pone.0124124 (PMC4390299; doi:10.1371/journal.pone.0124124)

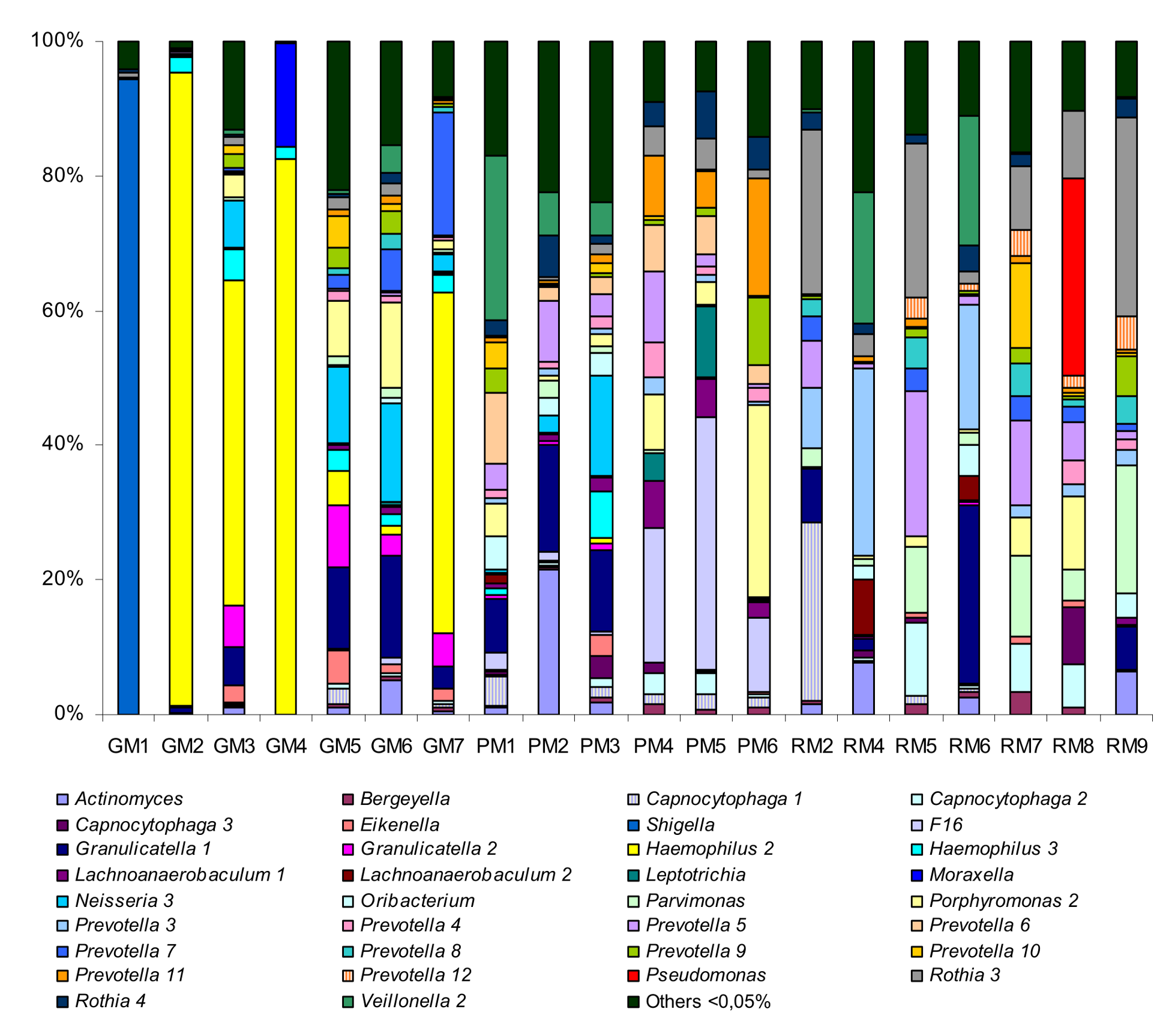

Supplement: S1 Fig — (TIF) [file pone.0124124.s002.tif]

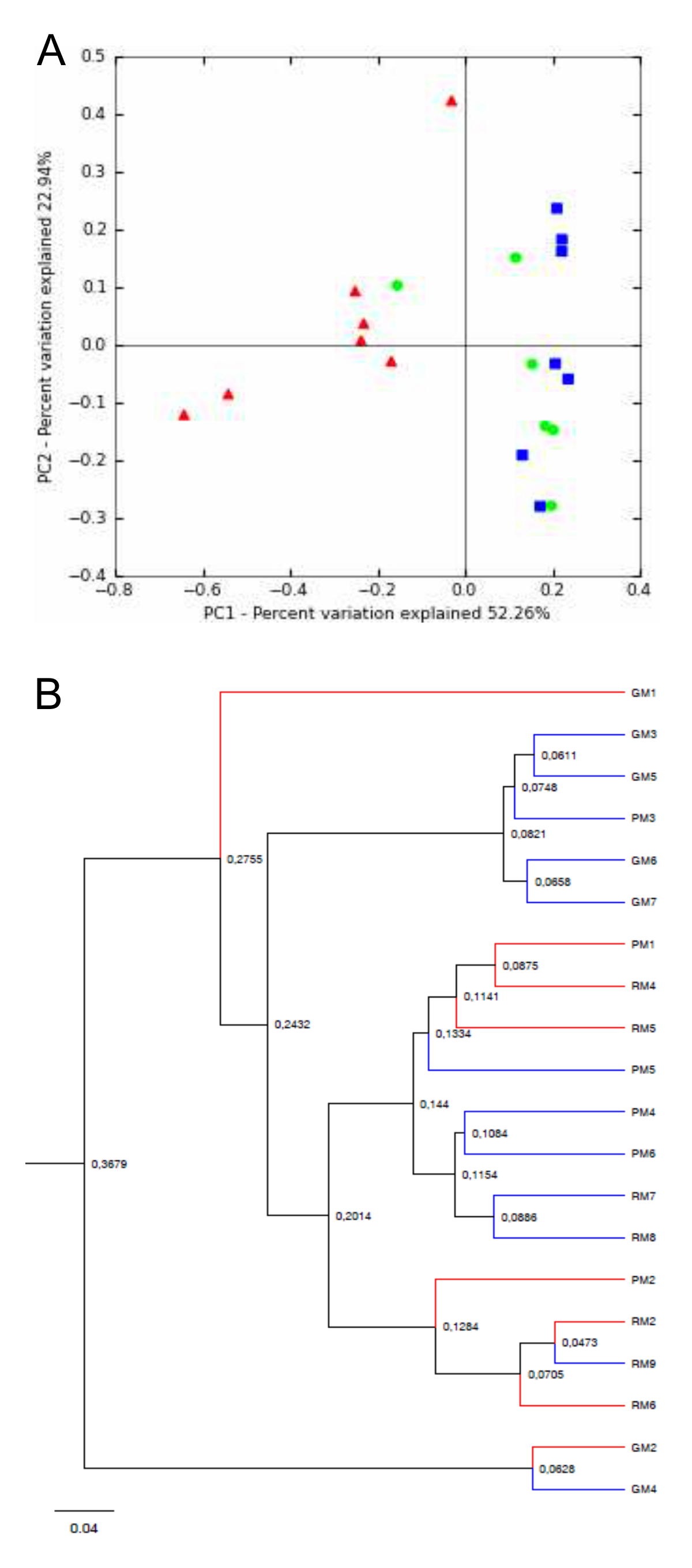

Supplement: S2 Fig — (TIF) [file pone.0124124.s003.tif]

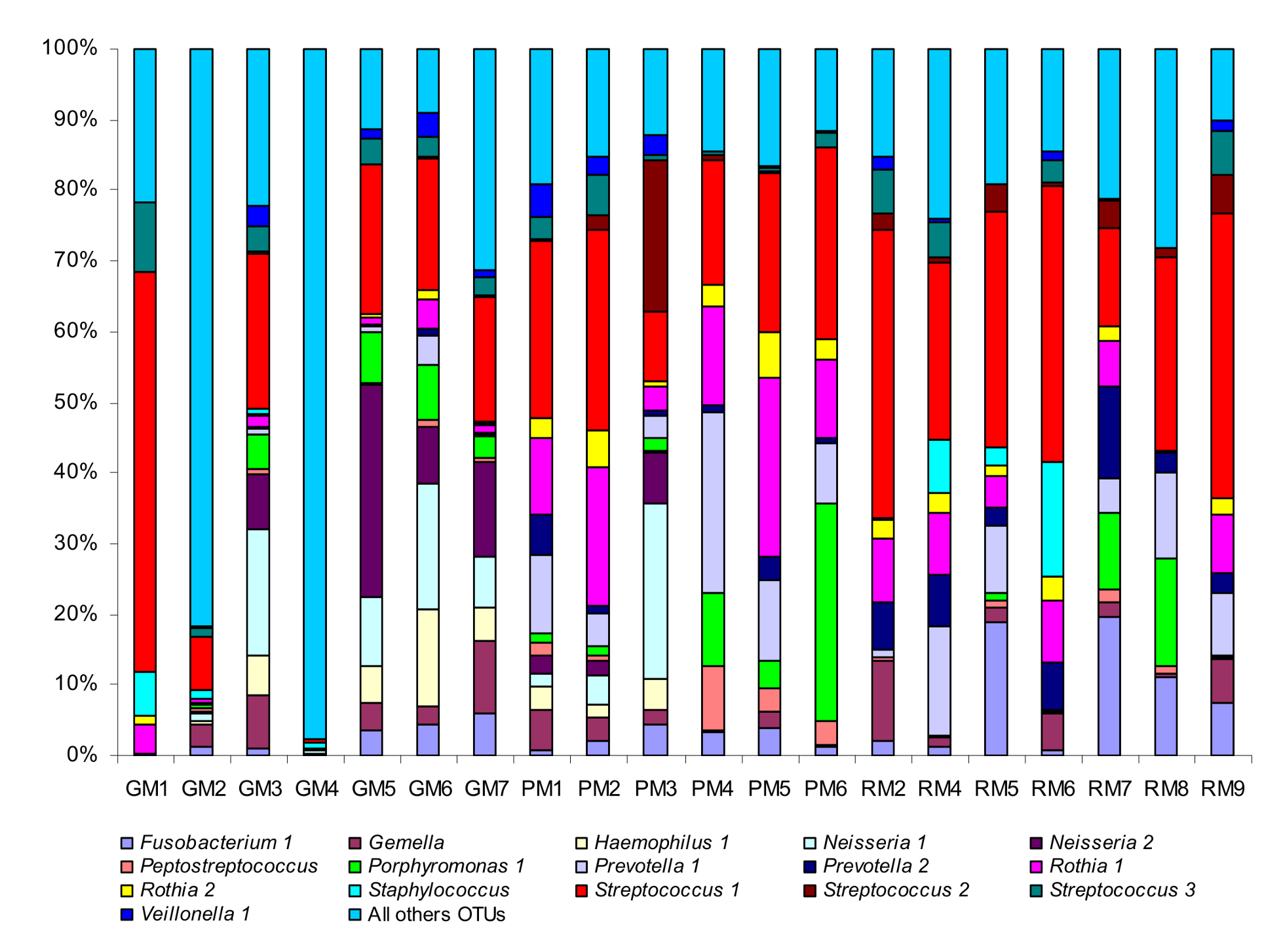

Supplement: S3 Fig — The very high RA of “All other OTUs” in samples GM2 and GM4 can be explained by the high prevalence of Haemophilus 2 (Haemophilus influenzae) in these samples (S1 Fig). (TIF) [file pone.0124124.s004.tif]

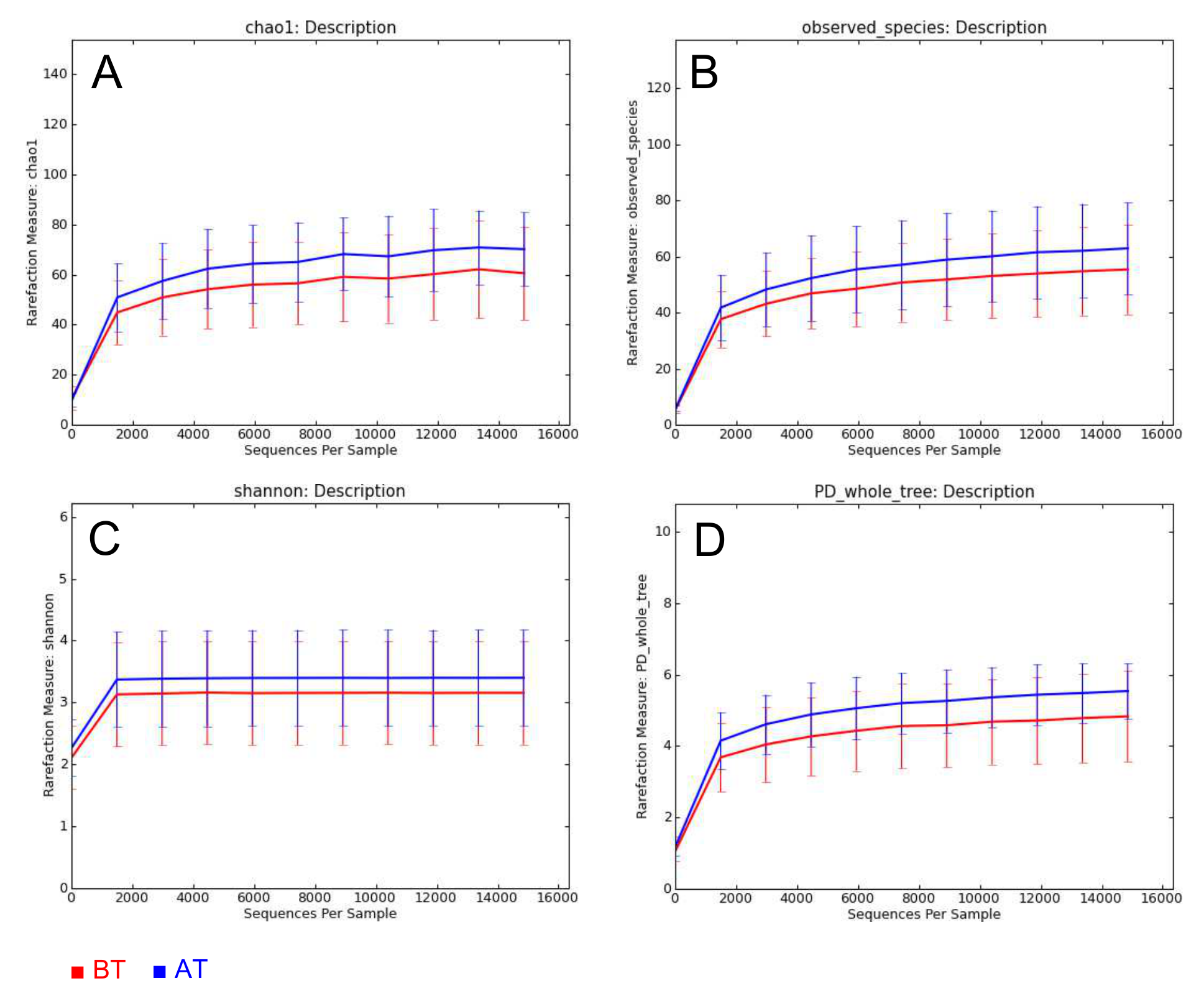

Supplement: S4 Fig — (TIF) [file pone.0124124.s005.tif]
