# Supplementary material for: Impact of the CFTR-Potentiator Ivacaftor on Airway Microbiota in Cystic Fibrosis Patients Carrying A G551D Mutation
Source: PLoS One. 2015 Apr 8;10(4):e0124124. doi: 10.1371/journal.pone.0124124 (PMC4390299; doi:10.1371/journal.pone.0124124)
Supplement: S1 Table — (PDF) [file pone.0124124.s006.pdf]

S1 Table. Coordinates and Cosine square (Cos^2) of variables on F1 and F2 axes.

	Coordinates		Cos²		
**Variables***	**F1**	**F2**	**F1**	**F2**	**F1 + F2**
Ge	-0.47	**0.59**	0.22	0.34	0.56
Ha1	**-0.65**	**0.67**	0.43	0.45	0.87
Sta	**-0.51**	**-0.57**	0.26	0.33	0.58
Pre1	**0.74**	0.37	0.54	0.14	0.68
Str1	0.16	-0.04	0.02	0.00	0.03
Pept	**0.78**	0.18	0.60	0.03	0.64
Str2	**0.54**	0.48	0.29	0.23	0.53
Neis1	**-0.65**	**0.69**	0.43	0.47	0.90
Fus1	0.44	0.47	0.19	0.22	0.42
Rot1	**0.56**	0.21	0.31	0.04	0.35
Vei1	-0.34	**0.89**	0.11	0.79	0.90
Por1	**0.62**	0.28	0.38	0.08	0.46
Neis2	**-0.70**	**0.67**	0.50	0.45	0.94
Pre2	**0.90**	0.29	0.81	0.09	0.89
Rot2	**0.52**	0.27	0.27	0.07	0.35
Str3	**-0.57**	0.28	0.32	0.08	0.40
Other	-0.27	**-0.62**	0.07	0.38	0.45
qPCR	**-0.63**	0.07	0.39	0.00	0.40
FEV1	**0.72**	0.15	0.51	0.02	0.53
Chao1	-0.32	0.46	0.10	0.21	0.32
ObsSp	-0.34	**0.56**	0.11	0.32	0.43
Shan	0.18	**0.68**	0.03	0.47	0.50
PDwt	-0.10	**0.50**	0.01	0.25	0.26

*Relative abundance of:
Ge: *Gemella*
Ha1: *Haemophilus*1
Sta: *Staphylococcus aureus*
Pre1: *Prevotella* 1
Str1: *Streptococcus* 1 (*S. mitis* group)
Pept: *Peptostreptococcus*
Str2: *Streptococcus* 2 (*S. salivarius* group)
Neis1: *Neisseria* 1
Fus1: *Fusobacterium* 1
Rot1: *Rothia* 1
Vei1: *Veillonella* 1
Por1: *Pophyromonas* 1
Neis2: *Neisseria* 2
Pre2: *Prevotella* 2
Rot2: *Rothia* 2
Str3: *Streptococcus* 3 (*S. anginosus* group)
Other: OTUs not included in core microbiota
ObsSp: Observed species
Shan: Shannon
PDwt: Phylogenetic diversity whole tree
