# Supplementary material for: Impact of the CFTR-Potentiator Ivacaftor on Airway Microbiota in Cystic Fibrosis Patients Carrying A G551D Mutation
Source: PLoS One. 2015 Apr 8;10(4):e0124124. doi: 10.1371/journal.pone.0124124 (PMC4390299; doi:10.1371/journal.pone.0124124)
Supplement: S2 Table — (PDF) [file pone.0124124.s007.pdf]

S2 Table. Quantitative variables used for statistical analysis and p-values on Colin White test for all samples, comparing the BT (before treatment) and AT (after treatment) groups for relative abundance of OTUs.

Sample	Group	qPCR	Ge	Ha1	Sta	Pre1	Str1	Pept	Str2	Neis1	Fus1	Rot1	Vei1	Por1	Neis2	Pre2	Rot2	Str3	Other	Chao1	ObsSp	Shan	PDwt
**GM1**	BT	9.11	57	0	935	0	8397	0	21	0	0	608	0	0	0	0	164	1451	3222	18	18.0	1.9	1.713
**GM2**	BT	9.67	463	74	193	4	1097	71	20	140	207	71	36	86	51	6	29	194	12113	84.98	73.1	1.7	5.989
**PM1**	BT	9.09	841	513	1	838	3699	262	49	259	119	1621	687	175	397	1647	445	449	2853	68.52	67.3	4.1	5.433
**PM2**	BT	8.94	497	277	0	184	4236	134	282	617	296	2920	401	163	303	689	752	852	2252	72.21	65.2	3.8	5.487
**RM2**	BT	8.63	1694	0	50	1001	6053	68	379	0	301	1358	247	0	0	146	365	915	2278	50.73	47.3	3.3	4.476
**RM4**	BT	8.11	194	0	1129	1081	3714	18	118	0	173	1284	98	37	8	2298	422	724	3557	68.93	60.4	3.8	5.191
**RM5**	BT	8.16	305	0	376	376	4970	175	554	0	2798	658	9	134	0	1424	241	3	2832	72.43	57.5	3.5	5.637
**RM6**	BT	8.47	767	0	2432	1007	5788	0	76	2	110	1315	190	4	24	47	482	475	2136	55.41	55.0	3.2	4.664
**GM3**	AT	9.53	1091	853	83	57	3292	101	35	2661	168	224	425	749	1139	95	54	531	3297	92.71	84.9	3.9	6.39
**GM4**	AT	9.30	23	19	101	1	65	3	0	54	15	6	14	38	8	1	1	14	14492	61.29	38.1	1.0	3.883
**GM5**	AT	9.39	577	762	4	23	3137	42	23	1455	540	188	193	1100	4448	94	55	527	1687	78	75.0	3.5	5.788
**GM6**	AT	9.60	373	2054	10	172	2758	131	38	2646	650	599	496	1172	1191	610	188	412	1355	82.65	76.1	3.8	6.038
**GM7**	AT	9.28	1533	706	41	43	2607	73	13	1059	867	136	152	482	2010	39	51	386	4657	95.24	91.6	3.9	7.197
**PM3**	AT	9.04	305	663	0	139	1461	39	3171	3685	641	492	412	277	1067	444	119	127	1813	82.04	77.2	3.8	5.969
**PM4**	AT	8.97	45	0	4	171	2589	1358	115	0	489	2065	10	1538	0	3779	462	87	2143	52.45	49.1	3.5	4.921
**PM5**	AT	8.53	341	0	1	481	3336	512	32	0	564	3749	32	561	0	1723	980	82	2461	65.2	55.2	3.5	5.236
**PM6**	AT	9.61	42	0	1	114	4005	511	18	0	193	1654	29	4556	0	1264	434	299	1735	65.3	48.1	3.1	4.923
**RM7**	AT	8.26	302	0	8	1886	2052	254	565	0	2918	994	59	1623	0	752	300	1	3141	60.71	55.8	4.0	5.436
**RM8**	AT	7.99	64	0	44	406	4074	165	167	0	1654	0	11	2271	0	1812	0	0	4187	57.47	53.6	3.6	5.643
**RM9**	AT	8.75	944	4	2	420	5979	73	813	0	1106	1223	219	1	0	1288	368	922	1493	54.35	51.5	3.2	4.89
**P-value**		**>0.05**	**>0.05**	**>0.05**	**>0.05**	**>0.05**	**<0.05**	**>0.05**	**>0.05**	**>0.05**	**>0.05**	**>0.05**	**>0.05**	**<0.01**	**>0.05**	**>0.05**	**>0.05**	**>0.05**	**>0.05**	**>0.05**	**>0.05**	**>0.05**	**>0.05**
**Concl.**		**NS**	**NS**	**NS**	**NS**	**NS**	*****	**NS**	**NS**	**NS**	**NS**	**NS**	**NS**	******	**NS**	**NS**	**NS**	**NS**	**NS**	**NS**	**NS**	**NS**	**NS**

RA: Relative abundance
Concl. : conclusion
qPCR: log10 rRNA gene copies/mL
Ge: *Gemella* RA
Ha1: *Haemophilus*1 RA
Sta: *Staphylococcus aureus* RA
Pre1: *Prevotella* 1 RA
Str1: *Streptococcus* 1(S. mitis group)
Pept: *Peptostreptococcus* RA
Str2: *Streptococcus* 2 (*S. salivarius* group)
Neis1: *Neisseria* 1 RA
Fus1: *Fusobacterium* 1 RA
Rot1: *Rothia* 1 RA
Vei1: *Veillonella* 1 RA
Por1: *Pophyromonas* 1 RA
Neis2: *Neisseria* 2 RA
Pre2: *Prevotella* 2 RA
Rot2: *Rothia* 2 RA
Str3: *Streptococcus* 3 (*S. anginosus* group)
Other: OTUs out of major core microbiota
ObsSp: Observed species
Shan: Shannon
PDwt: Phylogenetic diversity whole tree

NS: Not significant
*: Significant
**: Highly significant
