# Supplementary material for: Impact of the CFTR-Potentiator Ivacaftor on Airway Microbiota in Cystic Fibrosis Patients Carrying A G551D Mutation
Source: PLoS One. 2015 Apr 8;10(4):e0124124. doi: 10.1371/journal.pone.0124124 (PMC4390299; doi:10.1371/journal.pone.0124124)
Supplement: S3 Table — (PDF) [file pone.0124124.s008.pdf]

S3 Table. Correlation (Spearman test) between relative abundance of OTUs belonging to the major core microbiota and qPCR results.

	Ge	Ha1	Sta	Pre1	Str1	Pept	Str2	Neis1	Fus1	Rot1	Vei1	Por1	Neis2	Pre2	Rot2	Str3	Oth	qPCR
Ge	1	**0.46**	-0.06	0.20	0.22	-0.12	0.14	0.41	0.09	0.03	**0.70**	-0.29	**0.46**	-0.22	0.06	**0.49**	-0.15	0.07
Ha1	**0.46**	1	-0.29	-0.44	-0.44	-0.14	-0.22	**0.93**	0.00	-0.41	**0.69**	0.15	**0.90**	-0.38	-0.38	0.20	-0.11	**0.64**
Sta	-0.06	-0.29	1	-0.04	0.19	**-0.55**	-0.21	-0.22	-0.31	-0.39	-0.37	**-0.46**	-0.10	-0.35	-0.34	0.08	**0.50**	-0.16
Pre1	0.20	-0.44	-0.04	1	0.34	0.25	**0.65**	-0.41	0.28	**0.53**	0.23	-0.05	-0.33	**0.61**	**0.58**	-0.03	-0.19	**-0.76**
Str1	0.22	-0.44	0.19	0.34	1	-0.10	0.29	**-0.47**	-0.11	0.40	-0.06	**-0.44**	-0.42	0.14	0.39	**0.56**	-0.26	-0.42
Pept	-0.12	-0.14	**-0.55**	0.25	-0.10	1	0.12	-0.25	0.44	**0.45**	-0.08	**0.68**	-0.30	**0.69**	0.37	**-0.46**	-0.16	-0.08
Str2	0.14	-0.22	-0.21	**0.65**	0.29	0.12	1	-0.20	**0.51**	0.27	0.23	-0.10	-0.29	**0.49**	0.28	0.01	-0.35	**-0.67**
Neis1	0.41	**0.93**	-0.22	-0.41	**-0.47**	-0.25	-0.20	1	-0.12	-0.38	**0.68**	0.13	**0.94**	**-0.46**	-0.33	0.13	-0.09	**0.57**
Fus1	0.09	0.00	-0.31	0.28	-0.11	0.44	**0.51**	-0.12	1	-0.12	-0.03	0.42	-0.15	0.39	-0.12	**-0.45**	-0.22	-0.36
Rot1	0.03	-0.41	-0.39	**0.53**	0.40	**0.45**	0.27	-0.38	-0.12	1	0.09	-0.05	-0.37	**0.51**	**0.97**	0.24	**-0.46**	-0.27
Vei1	**0.70**	**0.69**	-0.37	0.23	-0.06	-0.08	0.23	**0.68**	-0.03	0.09	1	-0.04	**0.66**	-0.08	0.13	0.43	-0.37	0.23
Por1	-0.29	0.15	**-0.46**	-0.05	**-0.44**	**0.68**	-0.10	0.13	0.42	-0.05	-0.04	1	0.10	0.38	-0.08	**-0.58**	-0.15	0.16
Neis2	**0.46**	**0.90**	-0.10	-0.33	-0.42	-0.30	-0.29	**0.94**	-0.15	-0.37	**0.66**	0.10	1	-0.41	-0.29	0.21	-0.04	**0.52**
Pre2	-0.22	-0.38	-0.35	**0.61**	0.14	**0.69**	**0.49**	**-0.46**	0.39	**0.51**	-0.08	0.38	-0.41	1	**0.50**	-0.28	-0.21	**-0.56**
Rot2	0.06	-0.38	-0.34	**0.58**	0.39	0.37	0.28	-0.33	-0.12	**0.97**	0.13	-0.08	-0.29	**0.50**	1	0.24	**-0.49**	-0.32
Str3	**0.49**	0.20	0.08	-0.03	**0.56**	**-0.46**	0.01	0.13	**-0.45**	0.24	0.43	**-0.58**	0.21	-0.28	0.24	1	-0.27	0.18
Oth	-0.15	-0.11	**0.50**	-0.19	-0.26	-0.16	-0.35	-0.09	-0.22	**-0.46**	-0.37	-0.15	-0.04	-0.21	**-0.49**	-0.27	1	-0.08
qPCR	0.07	**0.64**	-0.16	**-0.76**	-0.42	-0.08	**-0.67**	**0.57**	-0.36	-0.27	0.23	0.16	**0.52**	**-0.56**	-0.32	0.18	-0.08	1

Ge: *Gemella*
Ha1: *Haemophilus* 1
Sta: *Staphylococcus aureus*
Pre1: *Prevotella* 1
Str1: *Streptococcus* 1 (*S. mitis* group)
Pept: *Peptostreptococcus*
Str2: *Streptococcus* 2 (*S. salivarius* group)
Neis1: *Neisseria* 1
Fus1: *Fusobacterium* 1
Rot1: *Rothia* 1
Vei1: *Veillonella* 1
Por1: *Pophyromonas* 1
Neis2: *Neisseria*
Pre2: *Prevotella* 2
Rot2: *Rothia* 2
Str3: *Streptococcus* 3 (*S. anginosus* group)
Oth: OTUs out of the major core microbiota
