# Supplementary material for: Impact of the CFTR-Potentiator Ivacaftor on Airway Microbiota in Cystic Fibrosis Patients Carrying A G551D Mutation
Source: PLoS One. 2015 Apr 8;10(4):e0124124. doi: 10.1371/journal.pone.0124124 (PMC4390299; doi:10.1371/journal.pone.0124124)
Supplement: S4 Table — (PDF) [file pone.0124124.s009.pdf]

S4 Table. Uncorrected p-values associated with Spearman correlations between relative abundance of OTUs belonging to the major core microbiota and qPCR results.

/	Ge	Ha1	Sta	Pre1	Str1	Pept	Str2	Neis1	Fus1	Rot1	Vei1	Por1	Neis2	Pre2	Rot2	Str3	Oth	[illegible]
Ge	/	**0.04**	0.8018	0.3977	0.3404	0.6197	0.5585	0.0755	0.6934	0.8973	**0.0006**	0.2179	**0.0425**	0.3488	0.8035	**0.0265**	0.5309	[illegible]
Ha1	**0.04**	/	0.2184	0.0516	0.0521	0.5564	0.3403	**4.1e-09**	0.9839	0.0716	**0.0007**	0.5361	**9.10-8**	0.0976	0.0976	0.3978	0.6344	[illegible]
Sta	0.8018	0.2184	/	0.8697	0.432	**0.0112**	0.3842	0.3508	0.185	0.0895	0.1053	0.0395	0.664	0.1343	0.1484	0.7235	**0.0264**	[illegible]
Pre1	0.3977	0.0516	0.8697	/	0.1484	0.2851	**0.0018**	0.0734	0.2272	**0.0171**	0.3259	0.8304	0.1527	**0.0041**	**0.0069**	0.8949	0.4274	[illegible]
Str1	0.3404	0.0521	0.432	0.1484	/	0.6608	0.217	**0.0373**	0.645	0.0844	0.8011	0.0496	0.0639	0.5608	0.0896	**0.0106**	0.2709	[illegible]
Pept	0.6197	0.5564	**0.0112**	0.2851	0.6608	/	0.6109	0.2909	0.0539	**0.0453**	0.7239	**0.0011**	0.2014	**0.0008**	0.1043	**0.0418**	0.5058	[illegible]
Str2	0.5585	0.3403	0.3842	**0.0018**	0.217	0.6109	/	0.3959	**0.0208**	0.2567	0.3227	0.6701	0.2171	**0.0282**	0.2246	0.9799	0.1264	[illegible]
Neis1	0.0755	**4.10-9**	0.3508	0.0734	**0.0373**	0.2909	0.3959	/	0.6033	0.0946	**0.001**	0.5843	**7.10-10**	**0.0419**	0.153	0.5962	0.7085	[illegible]
Fus1	0.6934	0.9839	0.185	0.2272	0.645	0.0539	**0.0208**	0.6033	/	0.6045	0.8949	0.0649	0.5416	0.0856	0.609	**0.0467**	0.349	[illegible]
Rot1	0.8973	0.0716	0.0895	**0.0171**	0.0844	**0.0453**	0.2567	0.0946	0.6045	/	0.7052	0.8354	0.1069	**0.0217**	**7.10-13**	0.3038	**0.0435**	[illegible]
Vei1	**0.0006**	**0.0007**	0.1053	0.3259	0.8011	0.7239	0.3227	**0.001**	0.8949	0.7052	/	0.8749	**0.0015**	0.7527	0.5912	0.0565	0.1131	[illegible]
Por1	0.2179	0.5361	**0.0395**	0.8304	**0.0496**	**0.0011**	0.6701	0.5843	0.0649	0.8354	0.8749	/	0.6889	0.1	0.7288	**0.0071**	0.5288	[illegible]
Neis2	**0.0425**	**9.10-8**	0.664	0.1527	0.0639	0.2014	0.2171	**7.10-10**	0.5416	0.1069	**0.0015**	0.6889	/	0.0696	0.2094	0.3747	0.8637	[illegible]
Pre2	0.3488	0.0976	0.1343	**0.0041**	0.5608	**0.0008**	**0.0282**	**0.0419**	0.0856	**0.0217**	0.7527	0.1	0.0696	/	**0.0261**	0.2376	0.3695	[illegible]
Rot2	0.8035	0.0976	0.1484	**0.0069**	0.0896	0.1043	0.2246	0.153	0.609	**7.10-13**	0.5912	0.7288	0.2094	**0.0261**	/	0.3069	**0.0271**	[illegible]
Str3	**0.0265**	0.3978	0.7235	0.8949	**0.0106**	**0.0418**	0.9799	0.5962	**0.0467**	0.3038	0.0565	**0.0071**	0.3747	0.2376	0.3069	/	0.243	[illegible]
Oth	0.5309	0.6344	**0.0264**	0.4274	0.2709	0.5058	0.1264	0.7085	0.349	**0.0435**	0.1131	0.5288	0.8637	0.3695	**0.0271**	0.243	/	[illegible]
qPCR	0.7623	**0.0023**	0.5052	**10.10-5**	0.0666	0.7382	**0.0012**	**0.0081**	0.1196	0.2457	0.3291	0.4937	**0.0188**	**0.0108**	0.1623	0.4465	0.7527	

Significant correlations are represented in bold (uncorrected p-values, threshold: 0.05).

Ge: *Gemella*
Ha1: *Haemophilus* 1
Sta: *Staphylococcus aureus*
Pre1: *Prevotella* 1
Str1: *Streptococcus* 1 (Mitis group)
Pept: *Peptostreptococcus*
Str2: *Streptococcus* 2 (Salivarius group)
Neis1: *Neisseria* 1
Fus1: *Fusobacterium* 1
Rot1: *Rothia* 1
Vei1: *Veillonella* 1
Por1: *Pophyromonas* 1
Neis2: *Neisseria* 2
Pre2: *Prevotella* 2
Rot2: *Rothia* 2
Str3: *Streptococcus* 3
Oth: OTUs out of the major core microbiota
